# Supplementary material for: Dual-mode harvest solar energy for photothermal Cu2-xSe biomineralization and seawater desalination by biotic-abiotic hybrid
Source: Nat Commun. 2024 May 22;15:4365. doi: 10.1038/s41467-024-48660-z (PMC11111681; doi:10.1038/s41467-024-48660-z)
Supplement: Supplementary file 1 — Supplementary Information [file 41467_2024_48660_MOESM1_ESM.pdf]

Dual-mode harvest solar energy for photothermal $Cu_{2-x}Se$ biomineralization and seawater desalination by biotic-abiotic hybrid

Gong *et al.*

Supplementary Method 1. Characterization of the *S. oneidensis*-Se^0 NPs and *S. oneidensis*-$Cu_{2-x}Se$ NPs

The morphology was investigated by field emission scanning electron microscopy (SEM) (JSM-6700F, JEOL Co., Japan) equipped with 5 kV. For SEM observation, hybrids were centrifuged and washed three times with phosphate-buffered saline (PBS) buffer (pH = 7.2), and then fixed with 2.5% glutaraldehyde overnight at 4 ^oC. The fixed cells were centrifuged and washed three times with PBS buffer (pH = 7.2). Subsequently, the washed cells were dehydrated using gradient ethanol concentration (30%, 50%, 70%, 80%, 90%, 93%, 95%, 97%, 100%) and immersed at each concentration for 20 min. The dehydrated cells were dripped on a silicon chip for SEM analyses. Raman spectra and mapping of the sample were obtained using a micro-Raman spectrometer (LabRAM HR Evolution, Horiba Co., Japan) excited by a 532 nm laser. The as-prepared hybrids were dripped on a tinfoil plate and then for measurements. For Raman mapping, 50 × objective was used. The used laser power was 10 mW, and the Raman shift range was 150-600 cm^{-1}. The exposure times was 5 s. The number of accumulations was two.

The biotic-abiotic hybrids were freeze-dried (FD-1C-50, Beijing Boyikang Experimental Instrument Co., Ltd, China) and ground into a powder for further X-ray diffractometer (XRD, SmartLab, Rigaku corporation, Japan) and UV-vis diffuse reflectance spectrum (UV-vis-DRS, Shimadzu's SolidSpec-3700/3700DUV spectrophotometers) measurement. The UV-vis-DRS spectrum was collected in the 200-1000 nm range. The band gap of biogenic Se^0 NPs were determined by Tauc plots[1]:

$$(\alpha h v)^{1/n} = A(hv - E_g) \quad \textbf{(1)}$$

where A is a constant of proportionality, v is the photon's frequency, α is the absorption coefficient, h is Planck's constant, and E_g is the band gap. And n = 2 applies to the indirect band

gap semiconductor Se^0 NPs. Analyzing the UV-vis-DRS data, $(\alpha hv)^{1/n}$ and hv were calculated and plotted as $(\alpha hv)^{1/n}$ versus hv. Where $hv = hc/\lambda$, where c is the speed of light and λ is the corresponding wavelength. Extrapolate the straight line portion of the resulting graph to the abscissa axis, and the crossing point is the band gap of the biogenic Se^0 NPs.

For UV-visible-near-infrared spectrophotometer (UV-vis-NIR) measurement, the treated cells were collected by centrifugation (3743 g, 5 min) and washed with deionized water for three times. The collected precipitate was resuspended in deionized water for UV-vis-NIR detection (UV-Vis-NIR, Shimadzu's SolidSpec-3700/3700DUV spectrophotometers). The test range was 450-1300 nm.

For the transient photocurrent response experiment, 3 mg samples were dispersed in 1 mL solvent of isopropanol and water (volume ratio = 1:1) and ultrasonic for 30 min. Subsequently, adding 2% (volume ratio) Nafion and ultrasound for another 30 min. The mixtures were dropped on the conductive side of a 2 cm × 1 cm fluorine-doped tin oxide (FTO) glass and dried at room temperature. Photo-electrochemical analyses were measured using a three-electrode system, in which the modified FTO served as a working electrode, a Pt sheet worked as a counter electrode, and an Ag/AgCl acted as a reference electrode. The electrolyte was 50 mM sodium sulfate. The photocurrent (*i-t*) of the three-electrode system under light illumination was recorded using a CHI-660E electrochemical workstation (Chenhua Inc., Shanghai, China). The potential of the working electrode was maintained at -0.3 V (vs Ag/AgCl). Light source was a 300 W Xenon lamp (PLS-SXE300D, Beijing Pophile Technology Co., Ltd, China) equipped with a 420 nm cut filter.

For X-ray photoelectron spectroscopy (XPS) detection, *S. oneidensis*-$Cu_{2-x}Se$ NPs were collected and washed three times with deionized water. The washed *S. oneidensis*-$Cu_{2-x}Se$ NPs

were freeze-dried and then ground for XPS (Kratos Axis supra+, Shimadzu) measurement.

Supplementary Method 2. Selenite reduction analysis

Selenite reduction was monitored by detecting selenium in the supernatant using inductively coupled plasma mass spectrometry (ICP-MS, iCAP RQ, Thermo Fisher Scientific, USA). To quantify selenium content in the supernatant, biological samples were collected at the sampling time point and then centrifuged. Subsequently, the obtained supernatant was digested in a mixture of HNO_3 and $HClO_4$ (volume ratio = 4:1). The resulting digestive fluid was diluted to 5 mL with deionized water for further ICP-MS assay.

Supplementary Method 3. Se^0 NPs content assay

To quantify time-resolved Se^0 content, biological samples were collected at the sampling time point. The obtained samples were centrifuged and washed three times with deionized water. Subsequently, the washed precipitate was re-suspended in deionized water. The corresponding absorption at 550 nm wavelength was measured using UV-VIS spectroscopy (UV-2600, Shimadzu, Japan).

Supplementary Method 4. Measurement of precipitated Cu and Cu(I) concentrations

The Cu(ⅠI) or Cu(Ⅰ) concentration in the liquid phase was detected and calculated using bathocuproine sulfonic acid disodium salt (BCS) chromogenic methods. The relevant experiments were performed in an anaerobic workstation (Electrotek scientific limited, United Kingdom). All solvents were aerated with N_2 to make them anaerobic. Liquid-liquid extraction was required when detecting Cu(I) according to previous work[2]. The aqueous two-phase system was formed by mixing 0.4 mL sample, 0.1 mL trichloroacetic acid (10% dissolved in water), 2

mL glycine buffer solution (0.05 M, pH = 12.5), and 1 mL 2,2'-biquinoline (0.05% dissolved in N-pentanol) and then vortexed for 30 min. After static layering, the extracted Cu(II) was dissolved in the bottom layer.

The total copper in the supernatant or the extracted Cu(II) were quantified by a colorimetric method using BCS[3]. After the samples were taken at the sampling time point, the corresponding supernatant was obtained by centrifugation. The detected solutions were formed by mixing 0.5 mL sample (supernatant liquid or extracted solution), 1.6 mL hydroxylamine hydrochloride (60 g L^{-1}), 1 mL sodium citrate (200 g L^{-1}), and 1 mL bathocuproine solution (1.8 g L^{-1}) and then vortexed for 60 min. The obtained orange reaction solution was measured on a UV-Vis spectrophotometer (UV-2600, Shimadzu, Japan) at 480 nm. The aqueous phase Cu(I) content is obtained by subtracting Cu(II) in the extracted solution from the total copper in the supernatant. The precipitated Cu content equals to the initially added copper minus the total copper in the liquid phase at the sampling point.

Supplementary Method 5. Photothermal properties measurement

After the samples were taken at the sampling time point, the collected 1 mL samples were washed three times with ultrapure water and resuspended in 200 μL ultrapure water for photothermal properties detection. The 200 μL washed samples were put in EP tubes and irradiated for 6 min under 1064 nm laser (Inter-Diff Co., China) at 1 W/cm^{-2} of output power. The temperature was recorded by an infrared camera (ICI7320, Infrared Camera Inc., Beaumont, Texas, USA).

Supplementary Method 6. Mechanism of the light-boost $Cu_{2-x}Se$ NPs assembly

To uncover the abiotic process for $Cu_{2-x}Se$ NPs production, the *S. oneidensis*-Se^0 NPs hybrid was collected, washed and resuspended in saturated potassium chloride, and then treated with ultrasonic disruption (200 W, 4 s ultrasound with 6 s intervals, 200 repeats) to fragment the bacterium. The lysed hybrid was then transferred into the anaerobic tube and purged with 100% N_2 for 30 min. Then, adding 1 mM $CuCl_2$ or CuCl to the lysed hybrid for $Cu_{2-x}Se$ NPs synthesis. The above experiments were performed in an anaerobic workstation (Electrotek scientific limited, United Kingdom). CuCl stock solution was prepared by dissolving CuCl in oxygen-free saturated potassium chloride, which needs to be prepared freshly when it is used.

For the biotic reaction, *S. oneidensis*-Se^0 NPs hybrid was collected, washed and resuspended in SMB. The resuspended hybrid was treated with ultra-sonication treatment to lyse the bacterium. The inactivated hybrid was then transferred into the anaerobic tube and purged with 100% N_2 for 30 min. Subsequently, 1 mM EDTA-Cu(II) and different strains were injected into the lysed hybrid for $Cu_{2-x}Se$ NPs synthesis.

The samples of the experiments in Fig. 3B-3G were from six independent biological replicates. The control groups were the experiments that in the dark or made by WT strains.

Supplementary Method 7. Detection of lactate

Lactate was measured by high-performance liquid chromatography (HPLC, Agilent 1260 Infinity, Germany) equipped with carbohydrate columns (Agilent Hi-Plex PL1170-6830, Germany). The mobile phase is 5 mM H_2SO_4 at a flow rate of 0.5 mL/min. The lactate was detected with a differential refractive index detector. The samples were centrifuged at 10000 g for 5 min. The collected supernatant was filtered through a 0.22 μm membrane and then for HPLC detection.

Supplementary Method 8. Nitrate reduction mediated by *S. oneidensis*-Se^0 NPs

For nitrate reduction, the constructed *S. oneidensis*-Se^0 NPs were collected, washed and resuspended in the anaerobic tube containing a modified SMB medium. The concentration of resuspended cells was adjusted to an optical density of 1.0 at 600 nm. The modified SMB contains (per liter) 0.09 g KCl, 0.67 g $NaH_2PO_4.2H_2O$, 11.91 g 4-(2-hydroxyethyl)-1-piperazineethanesulfonic acid (HEPES)， 1 mM sodium nitrate and 20 mM acetate. The pH of the SMB was adjusted to 7.2. The anaerobic condition was prepared by purging with 100% N_2 for 30 min and sealed with rubber. The used illumination source was a collimated Xenon lamp (PLS-SXE300D, Beijing Pophile Technology Co., Ltd., China) with a 420 nm UV-cut filter. The reaction time was 10 h and the light intensity was 20 mW/cm^2. The concentrations of nitrate (NO_3^--N), nitrite (NO_2^--N) and ammonia (NH_4^+-N) were measured according to the standard methods[4]. The samples of the experiments were from at least three independent biological replicates.

Supplementary Method 9. Physicochemical properties of membranes

Contact angle (JC2000D1, Shanghai Zhongchen Digital Technology Equipment Co., Ltd, China), AFM (Bruker Dimension Icon, Germany), ATR-FTIR (Thermo Scientific, USA), SEM (JSM-6700F, JEOL Co., Japan) and Raman spectral analysis (LabRAM HR Evolution, Horiba Co., Japan) were used to characterize the physicochemical properties of membranes.

Supplementary Method 10. Structural, chemical and performance stabilities of membranes

To investigate the structural, chemical and performance stabilities of the evaporator membrane, membranes were irradiated under 1 solar light intensity for 10 h. Subsequently, the concentrations of copper in the solution were quantified by ICP-MS (iCAP RQ, Thermo Fisher Scientific, USA). For XPS detection, membranes were collected and washed three times with deionized water. The washed membranes were for XPS (Kratos Axis supra$^+$, Shimadzu, Japan) measurement. The solar evaporation performance of the membranes was measured according to the method described in the manuscript. The membranes of the same type were divided into different individuals of the same size and shape for separate photothermal distillation experiments. And solar evaporation performance experiments of different membranes were from at least three independent replicates in Fig. 5E-5F, Supplementary Fig. 13, 16, and 17.

Supplementary Method 11. Measurement of raw and distilled water quality

The concentrations of Na^+, K^+, Ca^{2+}, Mg^{2+}, B^{3+} and Cu^{2+} in the distilled water were quantified by ICP-MS (iCAP RQ, Thermo Fisher Scientific, USA). For TOC measurement, the collected samples were filtered through a 0.22 μm membrane and then mixed with 2 M HCl at a volume ratio of 20:1. The mixed solutions were used for TOC detection by total organic carbon analyzer (Multi N/C 2100 TOC, Analytik Jena, Germany). For protein detection, the collected samples were mixed with NaOH (final concentration, 1 mM). Then, the mixtures were treated at 95 °C for 10 minutes to extract the proteins. The suspensions were for protein detection by an enhanced BCA protein assay kit (P0009, Beyotime Biotechnology, Shanghai, China). The samples were derived from distilled water or raw water under independent photothermal distillation experiments, and they were at least three independent replicates.

Supplementary Method 12. Chemical synthesis and characterization of $Cu_{2-x}Se$ NPs

According to the previous $Cu_{2-x}Se$ NPs synthesis protocol[5], 55 mL deionized water and 16 mL polyvinylpyrrolidone K30 solution (PVP-K30, 5 mg mL^{-1}) were mixed evenly in a beaker. Subsequently, 1 mL selenium dioxide (SeO_2, 0.2 M) and 3 mL vitamin C (Vc, 0.4 M) solutions were added. After stirring for 15 minutes, the solution turned red, which is a characteristic of Se^0 NPs. A fresh mixture of 1 mL $CuSO_4 \cdot 5H_2O$ (0.4 M) and 4 mL Vc (0.4 M) was added to the above-obtained red Se solution. The obtained mixture was stirred at 30 °C for 8 h. The mixture turned from red to brown black, suggesting that PVP-modified $Cu_{2-x}Se$ NPs were synthesized. The chemically synthesized $Cu_{2-x}Se$ NPs were collected and washed with deionized water for 6 times. The washed $Cu_{2-x}Se$ NPs were freeze-dried for further membrane construction. The as-prepared $Cu_{2-x}Se$ NPs were dripped on a copper screen and then for TEM image (H-7650, Hitachi, Ltd., Japan). The as-prepared $Cu_{2-x}Se$ NPs were freeze-dried (FD-1C-50, Beijing Boyikang Experimental Instrument Co., Ltd, China) and ground into a powder for further X-ray diffractometer (XRD, SmartLab, Rigaku Corporation, Japan).

Supplementary Method 13. Fabrication of Δ*cym*A-NPs@MR-1@PVDF membrane (using Δ*cym*A strain as bio-nano- factory)

Biogenic $Cu_{2-x}Se$ NPs were synthesized by Δ*cym*A following the procedure in Supplementary Fig. 1B. The resulting Δ*cym*A-nanoparticles hybrid was collected, freeze-dried, ground, and then used for membrane preparation. The casting solution was prepared by mixing polyvinyl pyrrolidone (PVP) and N,N-dimethylformamide (DMF) according to Supplementary Table 3. The sample information was the same as for the other membrane groups described above.

Supplementary Method 14. Purification of biogenic $Cu_{2-x}Se$ NPs

S. oneidensis-$Cu_{2-x}Se$ samples were collected by centrifugation and washed three times with ultrapure water. After that, the washed samples were resuspended in 2% sodium dodecyl sulfate

(SDS) and then soaked in a 55°C water bath for 1 h. The precipitate was collected by centrifugation and washed three times with ultrapure water. Subsequently, the precipitate was resuspended in water and treated with ultrasonic disruption (200 W, 4 s ultrasound with 6 s intervals, 90 repeats) to fragment the bacterium. The fragmented samples were centrifugated to obtain the supernatant solution. The acquired supernatant solution was further digested with proteinase K (100 μg/mL) at 55 °C for 0.5 h. The digested solution was centrifugated and washed three times to obtain purified $Cu_{2-x}Se$ NPs. The purified $Cu_{2-x}Se$ NPs were used for UV-vis-NIR measurement.

Supplementary Method 15. Statistical analysis

All experiments were performed in at least three parallel groups, and the results are shown as means ± standard deviation (SD). Independent samples *t*-test and one-way analysis of variance (GraphPad Prism version 9.5) were used to test the significant differences between two groups of data and the significant differences between multiple groups of data, respectively. Bonferroni was applied when performing an analysis of variance. The level of difference depends on the calculated *p*-value, and $p < 0.05$ indicates a significant difference. All experiments were repeated at least three times independently with similar results.

Supplementary Table 1. The concentration of bio-transformed Cu by lysed hybrid co-incubated with different strains with sodium lactate supplementation and incubated for 5 h.

Groups	Strains	Light/Dark	Bio-transformed Cu concentration (mg/L)
L-WT	Wild type	Light	5.94
L-Δ	Δ*omc*AΔ*mtr*C	Light	3.05
D-WT	Wild type	Dark	1.80
D-Δ	Δ*omc*AΔ*mtr*C	Dark	1.92

The concentration of copper was detected in the groups with sodium lactate supplementation and incubated for 5 h. By calculating the difference in Cu conversion between the L-WT group and the D-WT group, the photocatalytic synthesis portion is about 4.14 mg/L. The difference in Cu conversion between the L-WT group and L-Δ is the MtrC/OmcA proteins-mediated intracellular photoelectron utilization, as marked as number 1 in Figure 4, which is 2.89 mg/L. Therefore, the OmcA/MtrC proteins-mediated intracellular photoelectron utilization is 70%, and the remaining 30% is extracellular photoelectron utilization.

Supplementary Table 2. The amount of $Cu_{2-x}Se$ NPs produced by *S. oneidensis*-Se^0 hybrid with sodium lactate supplementation and incubated for 5 h.

Groups	Consumed Lactate (mM)	$Cu_{2-x}Se$ NPs Synthesis					
		Precipitated Cu (mg)	Cu(I) Ratio (%)	Produced Cu_2Se (mg)	Produced CuSe (mg)	Produced $Cu_{2-x}Se$ NPs (mg)	Ratio: produced $Cu_{2-x}Se$ NPs / consumed lactate (mg /mM)
Light	1.03	12.78	78.15	16.19	6.26	22.45	21.80
Dark	0.86	5.84	82.61	7.82	2.28	10.10	11.74

The proportion of Cu(I) in $Cu_{2-x}Se$ NPs was obtained from XPS result (Supplementary Fig. 5B and 5E).

Supplementary Table 3. Polyvinylidene fluoride (PVDF) membrane composition.

Membrane	PVP (g)	DMF (g)	Bio-Cu_{2-x}Se NPs (g)	Chem-Cu_{2-x}Se NPs (g)	*S. oneidensis* (g)	PVDF (g)
PVDF membrane	1	85	0	0	0	15
Bio-Cu_{2-x}Se@MR-1@PVDF (using WT as bio-nano-factory)	1	75	10	0	0	15
Δ *cym*A-NPs@MR-1@PVDF (using Δ*cym*A as bio-nano-factory	1	75	10	0	0	15
Chem-Cu_{2-x}Se@MR-1@PVDF	1	75	0	1.55	8.45	15
Chem-Cu_{2-x}Se@PVDF	1	83.45	0	1.55	0	15

PVP refers to polyvinl pyrrolidone. DMF refers to N,N-dimethylformamide. Bio-Cu_{2-x}Se refers to the hybrid of *S. oneidensis* and biogenic Cu_{2-x}Se NPs . Chem-Cu_{2-x}Se NPs refers to the chemically synthesized Cu_{2-x}Se NPs.

Supplementary Table 4. Comparison of solar steam device.

Supporting material	Absorber	Classification	Synthesis	Cost	Stability	Evaporation rate (kg m^{-2} h^{-1})	Conversion efficiency (%)	Ref.
PPB	AuNPs	Plasmonic metal	Using MSA and TFA	high	Easily aggregated and fused together at high temperature	1.424	83	6
Paper substrate	Au NPs	Plasmonic metal	Using $HAuCl_4$, boil for 75 min	high	Easily aggregated and fused together at high temperature	-	77.8	7
PVA	RGO	Carbonaceous	Using H_2SO_4, $KMnO_4$, $N_2H_4 \cdot H_2O$, glutaraldehyde	high	-	2.5	95	8
GO/NFC	CNT/GO	Carbonaceous	Using H_2SO_4, $KMnO_4$, NaClO, 80 °C	high	-	1.25	85.6	9
MCE	RGO	Carbonaceous	Using ascorbic acid, microwave reactor at 95 °C for 8 min	high	-	0.838	71.8	10
MGA	GO	Carbonaceous	Using H_2SO_4, $KMnO_4$, H_2O_2, 180 °C for 18 h	high	-	2.0	76.9	11
PS foam	RGO	Carbonaceous	Using H_2SO_4, $KMnO_4$, H_2O_2, TEOS, PS	high	A little salt can precipitate on the surface	1.31	83	12
GO foam	RGO	Carbonaceous	Using H_2SO_4, $KMnO_4$, laser	high	-	2.4	100	13
PS foam	CF	Carbonaceous	Using HNO_3 at 80 °C for 8 h	low	-	1.56	98.1	14
HNG	PPy	Carbonaceous	Using PVA, pyrrole, glutaraldehyde, APS	high	-	3.2	94	15
CW	TiN NPs	Plasmonic ceramics	Using APTES	high	-	-	> 80	16
PVDF	$Cu_{2-x}Se$	Plasmonic semiconductors	Photo-facilitated biosynthesis	low	Stability	1.44	90.55	This work

The above photothermal distillation membrane experiments were performed under 1 sun irradiation. PPB refers to p-phenylene benzobisoxazole. AuNPs refers to gold nanoparticle. MSA refers to methanesulfonic acid. TFA refers to trifluoroacetic acid. PVA refers to polyvinyl alcohol. GO refers to graphene oxide. NFC refers to nanofibrillated cellulose. CNT refers to carbon nanotube. MCE refers to mixed cellulose esters. RGO refers to reduced graphene oxide. MGA refers to modified graphene aerogel. PS refers to polystyrene. TEOS refers to tetraethyl orthosilicate. CF refers to carbon felt. HNG refers to hierarchically nanostructured gel. PPy refers to polypyrrole. PVA refers to polyvinyl alcohol. APS refers to ammonium persulfate. CW refers to ceramic fiber wool. TiN NPs refers to titanium nitride nanoparticles. APTES refers to 3-aminopropyl-triethoxysilane. PVDF refers to the poly(vinylidene fluoride) membrane. 3DHG refers to 3D hierarchical solar vapor generator.

Supplementary Table 5. Comparison of solar steam device based on Cu-composed nanoparticles.

Supporting material	Absorber	Synthesis of absorber	Synthesis conditions of absorber	Evaporation rate (kg m^{-2} h^{-1})	Conversion efficiency (%)	Membrane temperature (^{o}C)	Ref.
Filter paper	$Cu_{2-x}Se$@polydopamine	-	Using PVP, ascorbic acid, dopamine, H_2O_2	2.71	-	-	[17]
Glass microfiber	$Cu_{2-x}Se/Nb_2CT_x$	-	Using hydrazine, PVP, H_2O_2, HF aqueous solution, over one week	1.2	-	39.7	[18]
PVDF	Cu_9S_5	Hydrothermal	180 °C for several hours	1.173	80.2 ± 0.6	36.1	[19]
PVDF	CuS	Hydrothermal	180 °C for 18 h	1.43	90.4	38.5	[20]
MCE	CuS	Hydrothermal	140 °C for 12 h	1.12	80±2.5	42.8	[21]
SCM	CuS nanoflowers	Hydrothermal	120 °C for 18 h	1.09	68.6	35.2	[22]
Polyethylene	CuS	Hydrothermal	180 °C for 12 h	1.021	63.9	37.6	[23]
Cellulose hydrogel	CuS	Hydrothermal	120 °C for 12 h	2.2	87	-	[24]
PAAm-CMC	CuS	Hydrothermal	180 °C for 12 h	1.613	79	47.2	[25]
PVDF	$Cu_{2-x}Se$	Photo-facilitated biosynthesis	Room temperature	1.44	90.55	41.2	This work

The above photothermal distillation membrane experiments were performed under 1 sun irradiation. PVDF refers to the poly(vinylidene fluoride) membrane. PVP refers to poly(vinyl pyrrolidone). MCE refers to mixed cellulose ester membrane. SCM refers to semipermeable collodion membrane. PAAm-CMC refers to polyacrylamide (PAAm) and carboxymethyl cellulose (CMC).

Supplementary Table 6. Mechanism, regulation strategies and applications of biogenic Se^0 nanoparticles.

Organism	Mechanism	Regulation strategies	Applications	Ref.
Stenotrophomonas maltophilia SeITE02	Alcohol dehydrogenase homolog	-	-	[26]
Azospirillum thiophilum	Structural and compositional properties of Se^0 NPs.	-	-	[27]
Shewanella sp. HN-41	-	Reaction time, biomass, selenite concentration	-	[28]
Shewanella sp. HN-41	-	Reaction time and biomass	-	[29]
Pseudoalteromonas shioyasakiensis	-	-	Antimicrobial, antifouling and cytotoxic activities	[30]
S. oneidensis MR-1<br>*S. putrefaciens*	-	Adding riboflavin	-	[31]
S. oneidensis MR-1	-	Regulate extracellular electron transfer	-	[32]
S. oneidensis MR-1	Fumarate reductase FccA	-	-	[33]
S. oneidensis MR-1	-	-	*S. oneidensis*-Se^0 hybrid for solar energy conversion	This work

Supplementary Table 7. Biosynthesis and the corresponding applications of $Cu_{2-x}Se$ nanoparticles.

Organism	Precursors	Formation site	Electron source	Regulation strategies	Mtr pathway direction	Applications	Ref.
Pantoea agglomerans	SeO_3^{2-} & EDTA-Cu^{2+}	-	Metabolic electron	-	-	Photocatalytical degradation of methylene blue	[34]
S. onedensis	SeO_3^{2-} & Cu^{2+}	-	Metabolic electron	-	Normal	Photothermal antibacterial membrane	[35]
S. onedensis	SeO_3^{2-} & Cu^{2+}	-	Metabolic electron	Adding AQDS	Normal	-	[36]
S. onedensis	SeO_3^{2-} & Cu^{2+}	Periplasmic synthesis and then efflux out	Metabolic electron	Adjusting precursor concentration	Normal	Photothermal Therapy	[37]
S. onedensis	Se^0 & EDTA-Cu^{2+}	Extracellular fabrication	Metabolic electron & Photoelectron	Solar energy	Reversed	Solar Water Production	This work

EDTA refers to ethylenediaminetetraacetic acid tetrasodium salt dihydrate. AQDS refers to anthraquinone-2,6-disulfonate.

Supplementary Table 8. The direction of EET in *Shewanella oneidensis* MR-1 cells.

Electron source	Electron sink	Reaction site of electron acceptor	Electron flow	Ref.
Cathodic electrons<br>(-360 mV vs SHE)	Fumarate<br>(30 mV vs SHE)	Periplasm	Reverse MtrABC	38
Cathodic electrons<br>(-303 mV vs SHE)	Oxygen reduction,<br>NADH, $FMNH_2$<br>generation	Cytoplsm	Reverse MtrABC	39
Cathodic electrons<br>(-300 mV vs SHE)	2,3-butanediol	Cytoplsm	Reverse EET<br>Reverse NADH<br>dehydrogenases	40
Cathodic electrons<br>(-400 mV vs SHE)	Nitrate reduction	Periplasm	Reverse MtrABC	41
Photoelectron from<br>Cu_2O/RGO<br>(-510 mV vs SHE)	H_2	Periplasm	Reverse MtrABC	42
Photoelectron from CdS	Degradation of<br>trypan blue	Extracellular<br>space	Native EET	43
Photoelectron from CdS<br>(-680 mV vs SHE)	H_2	Periplasm	Reverse MtrABC	1
Chemical electron from<br>iron-containing metals	Nitrate reduction	Periplasm	Reverse MtrABC	44
Photoelectron from Se^0<br>(-736 mV vs SHE)	Cu(II) reduction<br>$Cu_{2-x}Se$ NPs<br>formation	Periplasm	Reverse MtrABC	This<br>work

EET refers to extracellular electron transfer. refers to anthraquinone-2,6-disulfonate. RGO refers to reduced graphene oxide.

Supplementary Table 9. Photo-induced biosynthesis of nanoparticles.

Organism	Mechanism for photo-induced biosynthesis	Nanoparticles synthesis	Ref.
S. oneidensis MR-1	Certain active groups and extracellular polymeric substances (EPS)	Gold NPs	[45]
Eucalyptus	-	Gold NPs	[46]
Lolium perenne	-	CdS_xSe_{1-x} quantum dots	[47]
Chlorella	Electrons from photosynthetic chain	Gold NPs	[48]
Phormidium ambiguum *Desertifilum tharense*	-	Silver NPs	[49]
S. oneidensis MR-1	Bacterium uses photogenerated electrons from illuminated Se^0 semiconductor	$Cu_{2-x}Se$ NPs	This work

Supplementary Table 10. Comparison of whole-cell biotic-abiotic hybrid systems for solar energy conversion.

Living organism	Materials	Synthesis methods	Location of semiconductor	Photoelectron transport to Product	Photoelectron utilization site	Products	Ref.
Engineered *Saccharomyces cerevisiae*	InP	Chemical	On cell surface	One pathway	Cytoplasm	Shikimic acid	50
Moorella thermoacetica	Gold nanoclusters	Chemical	Cytoplasm	One pathway	Cytoplasm	Acetic acid	51
M. thermoacetica	CdS	Biosynthesis	On cell surface	One pathway	Cytoplasm	Acetic acid	52
Escherichia coli	CdS nanocluster	Biosynthesis	Periplasm	One pathway	Cytoplasm	Malate	53
Methanosarcina barkeri	NiCu@CdS	Chemical	On cell surface	One pathway	Cytoplasm	Methane	54
Engineered *E. coli*	CdS	Biosynthesis	On cell surface	One pathway	Cytoplasm	Hydrogen	55
Desulfovibrio desulfuricans	CdS	Biosynthesis	On cell surface	One pathway	-	Hydrogen	56
S. oneidensis MR-1	$CuInS_2$/ZnS QDs	Chemical	Periplasm	One pathway	Cytoplasm	Hydrogen	57
S. oneidensis MR-1	RGO and Cu_2O	Chemical	Around cell	One pathway	Periplasm	Hydrogen	42
S. oneidensis MR-1	CdS	Biosynthesis	On cell surface	One pathway	Periplasm	Hydrogen	1
S. oneidensis MR-1	Se^0	Biosynthesis	On cell surface & Extracellular	Dual-Mode	Periplasm & Extracellular	$Cu_{2-x}Se$ NPs	This work

RGO refers to reduced graphene oxide. QDs refers to quantum dots.

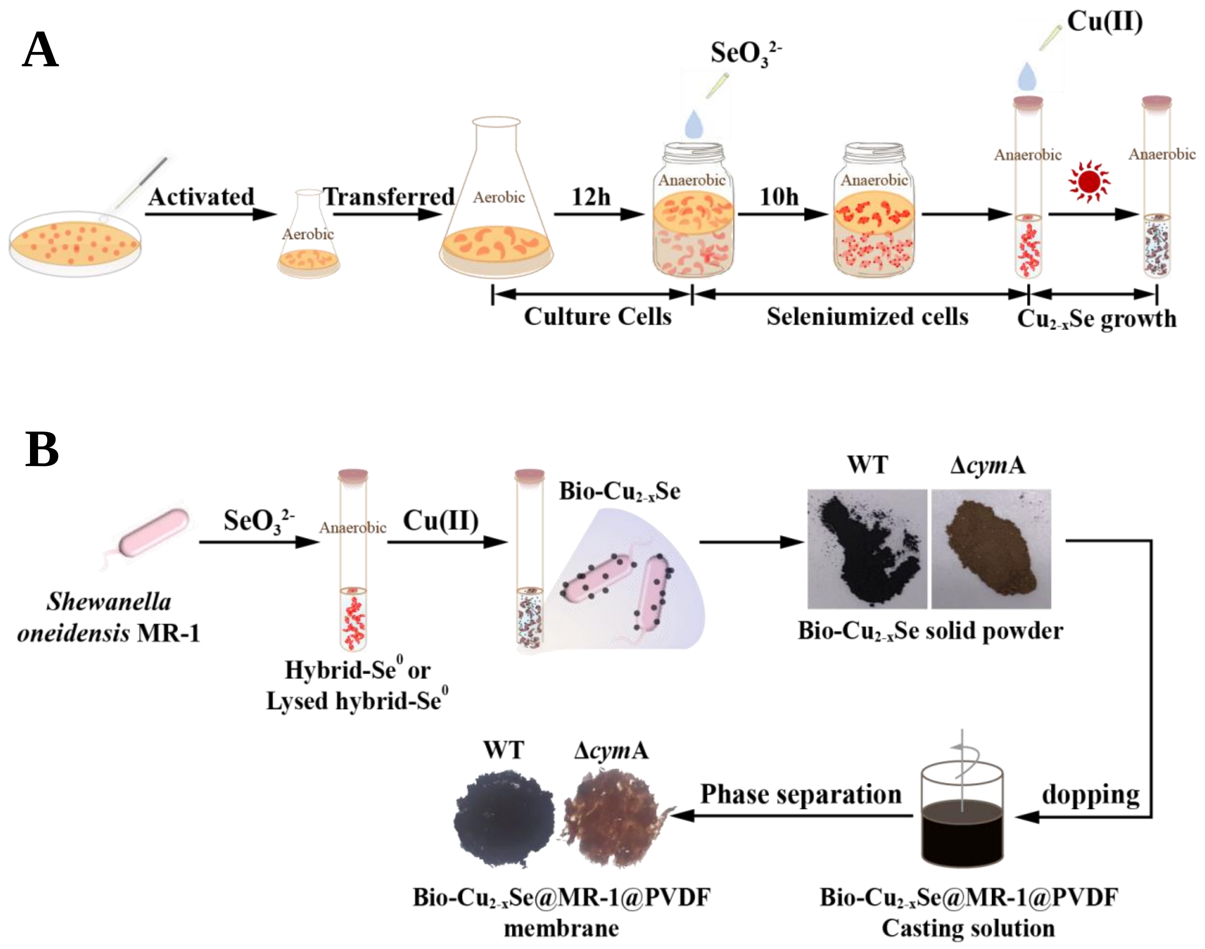

Supplementary Figure 1. Schematic diagram of the experimental procedures. (**A**) Biosynthesis of $Cu_{2-x}Se$ NPs with illumination by *S. oneidensis*-Se^0 hybrid system. (**B**) Preparation of Bio-$Cu_{2-x}Se$@MR-1@PVDF membrane (using WT as bio-nano-factory) or Δ*cym*A-NPs@MR-1@PVDF membrane (using Δ*cym*A as bio-nano-factory).

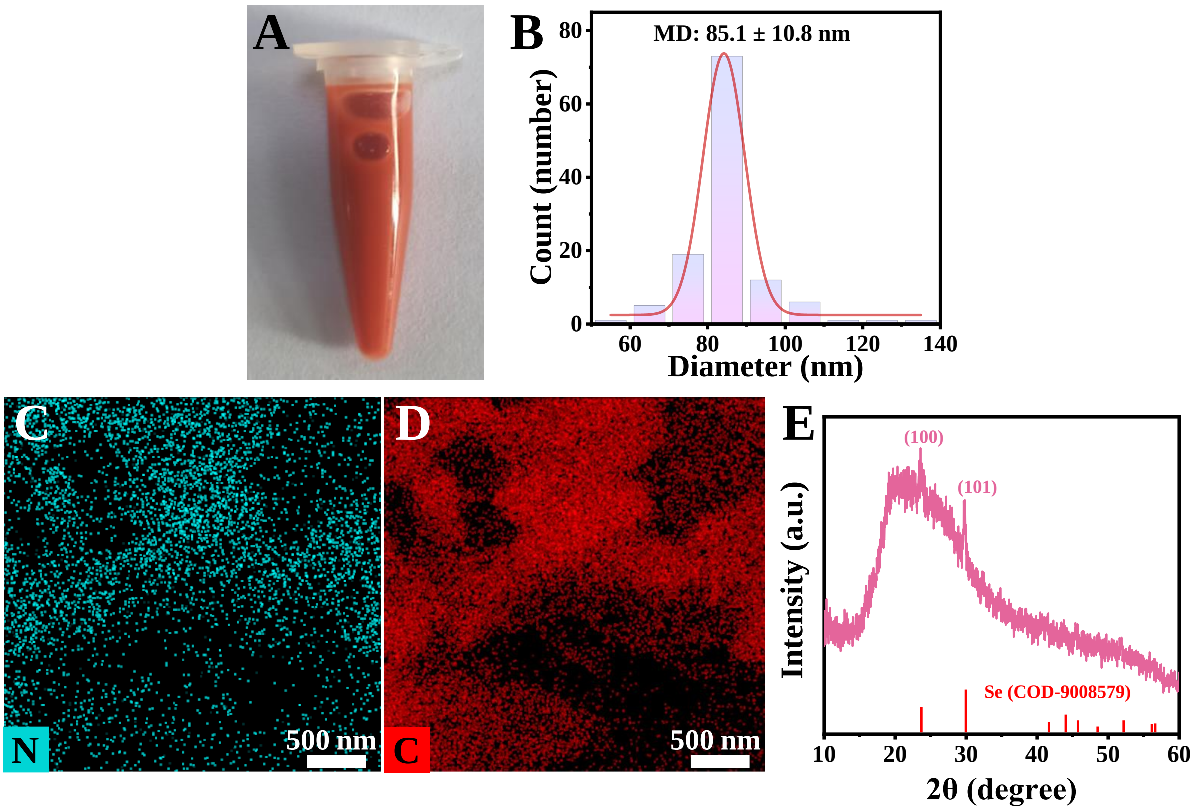

Supplementary Figure 2. Characteristics of the constructed *S. oneidensis*-Se^0 hybrid. (**A**) Optical images of *S. oneidensis*-Se^0 hybrid. (**B**) Average particle size of Se nanoparticles. The corresponding EDS mapping images of (**C**) N and (**D**) C elements of the biosynthesized *S. oneidensis*-Se^0 hybrid in Figure 1B. (**E**) XRD pattern of the formed *S. oneidensis*-Se^0 hybrid and standard Se (COD-9008579).

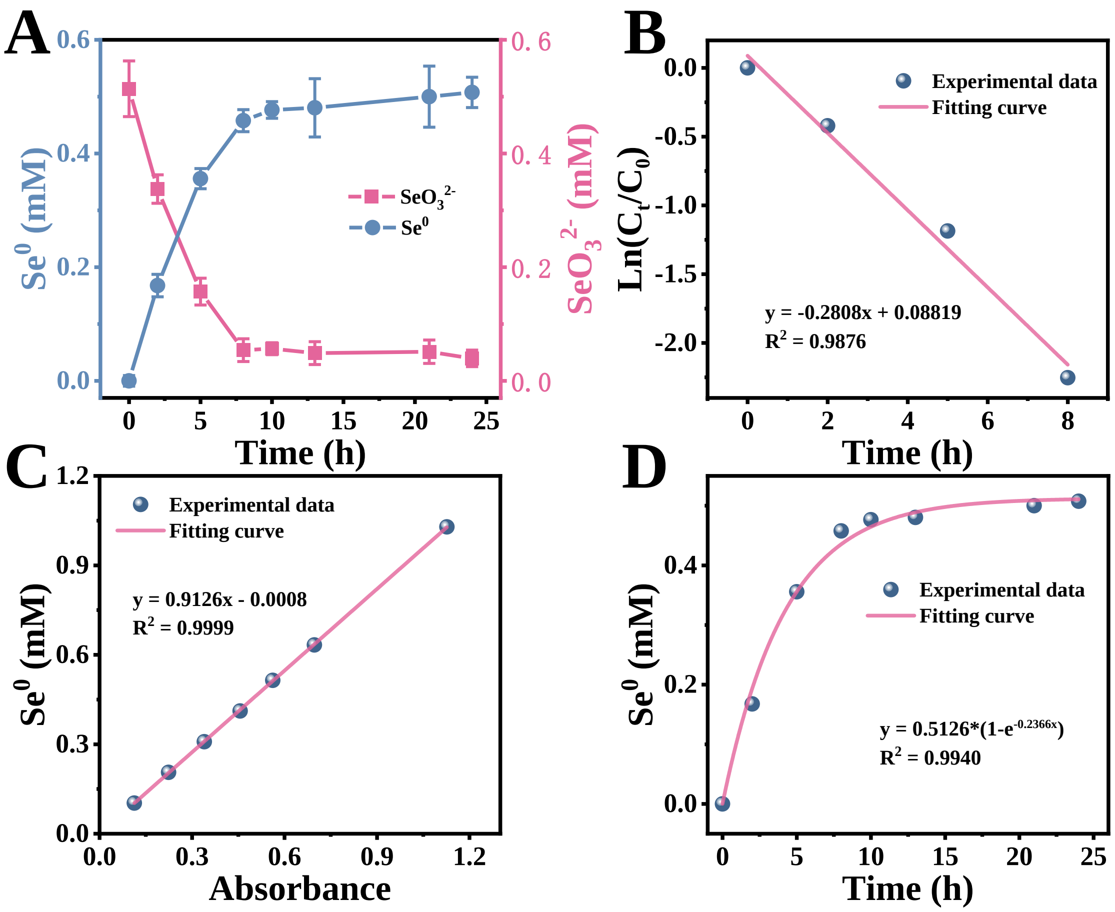

Supplementary Figure 3. The dynamics of selenite reduction and Se^0 NPs formation. (**A**) Time-resolved selenite reduction and Se^0 NPs formation concentration by *S. oneidensis* MR-1. (**B**) Kinetic curve of selenite reduction (R^2 = 0.9876). (**C**) A linear relationship (R^2 = 0.9999) between Se^0 NPs concentration and absorbance at 550 nm. (**D**) Kinetic curve of Se^0 NPs formation (R^2 = 0.9940). The above experiments were performed in LB mediums. The data points represented in (**A**) represent three (*n* = 3) independent experiments for each experimental group and are displayed as mean ± standard deviation (SD).

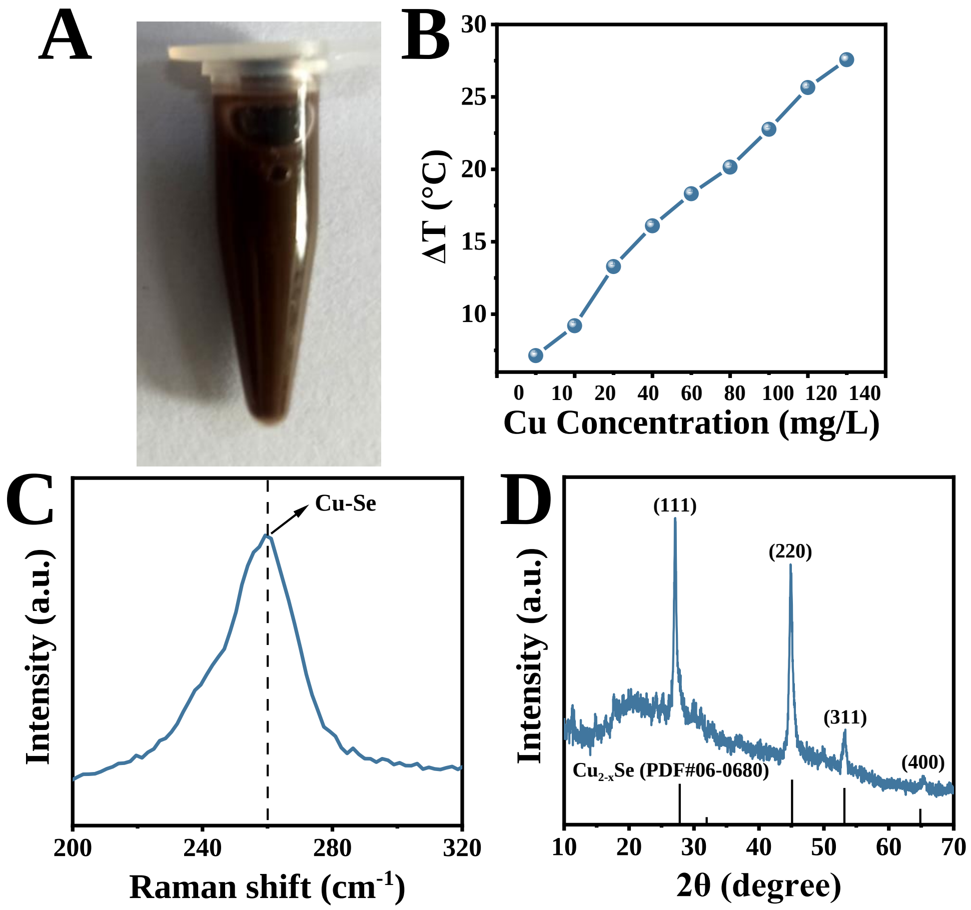

Supplementary Figure 4. Characteristics of the biogenic $Cu_{2-x}Se$ NPs. (**A**) Optical images of *S. oneidensis*-$Cu_{2-x}Se$ NPs. (**B**) Temperature rise (ΔT) of *S. oneidensis*-$Cu_{2-x}Se$ solution at gradient Cu concentrations between 0 and 140 mg/L. (**C**) In-situ micro-Raman spectrum of *S. oneidensis*-$Cu_{2-x}Se$ NPs. (**D**) XRD patterns of the formed *S. oneidensis*-$Cu_{2-x}Se$ NPs and standard $Cu_{2-x}Se$ (PDF#06-0680). The data points represented in (**B**) represent six (n = 6) repeated experiments for each experimental group and are displayed as mean ± standard deviation (SD).

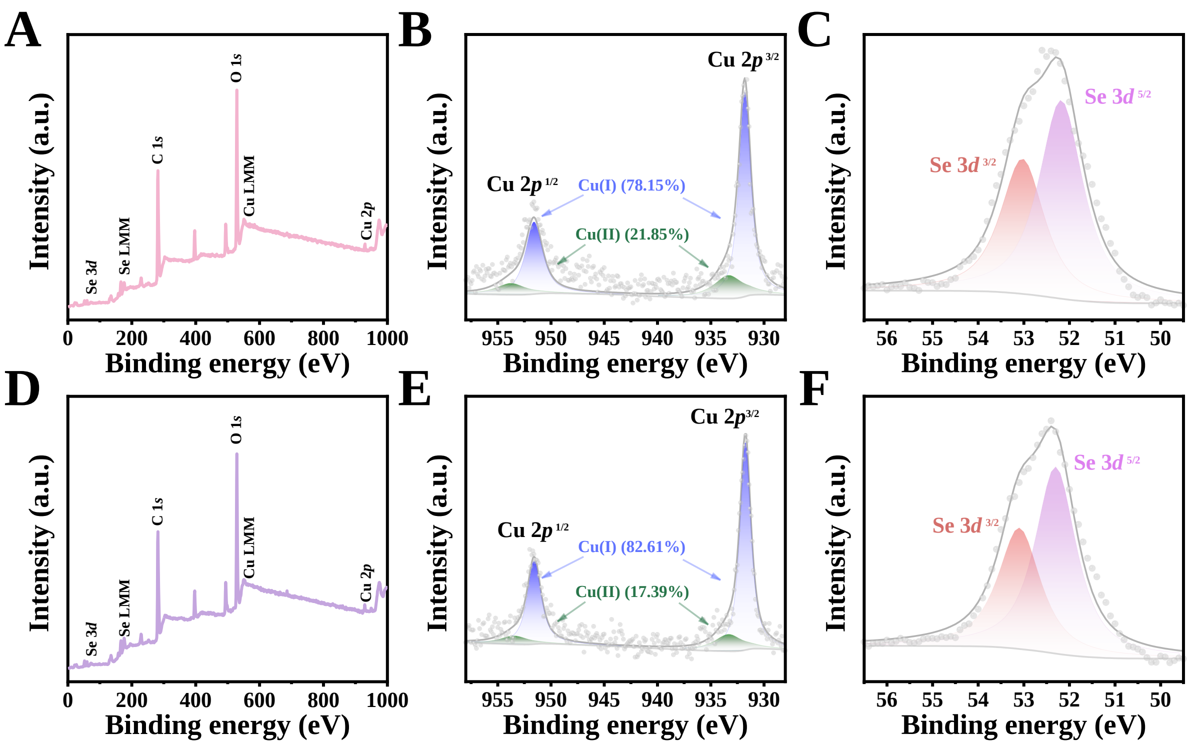

Supplementary Figure 5. XPS results of the biogenic $Cu_{2-x}Se$ NPs by *S. oneidensis*-Se^0 hybrid (A-C) with / (D-F) without illumination for 5 h. (**A, D**) XPS survey spectrum, (**B, E**) Cu 2*p* spectrum and (**C, F**) Se 3*d* spectrum.

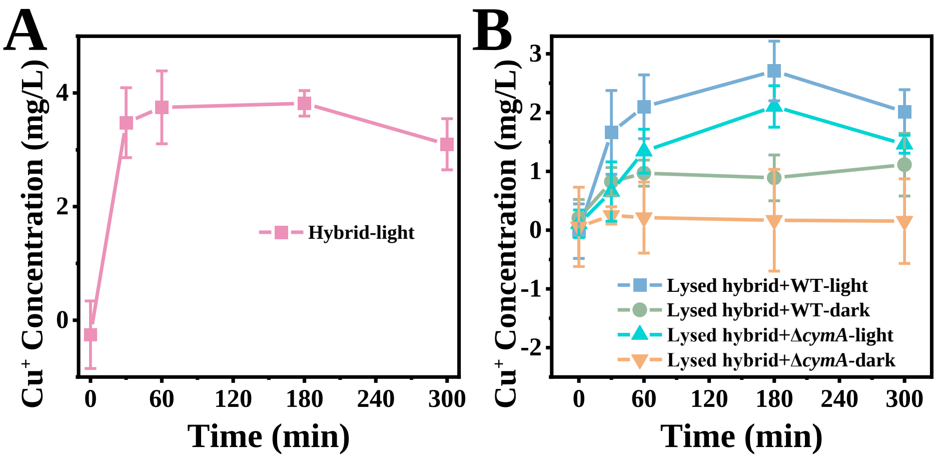

Supplementary Figure 6. Time-resolved aqueous Cu(I) concentration during the $Cu_{2-x}Se$ NPs assembly. (**A**) The *S. oneidensis*-Se^0 hybrid with Cu(II) under light condition. (**B**) The lysed hybrid co-incubated with WT/Δ*cym*A strains and Cu(II) with/without illumination. The above experiments were performed in the mineral salt medium with 20 mM sodium lactate. The data points represent three (*n* = 3) independent experiments for each experimental group and are displayed as mean ± standard deviation (SD).

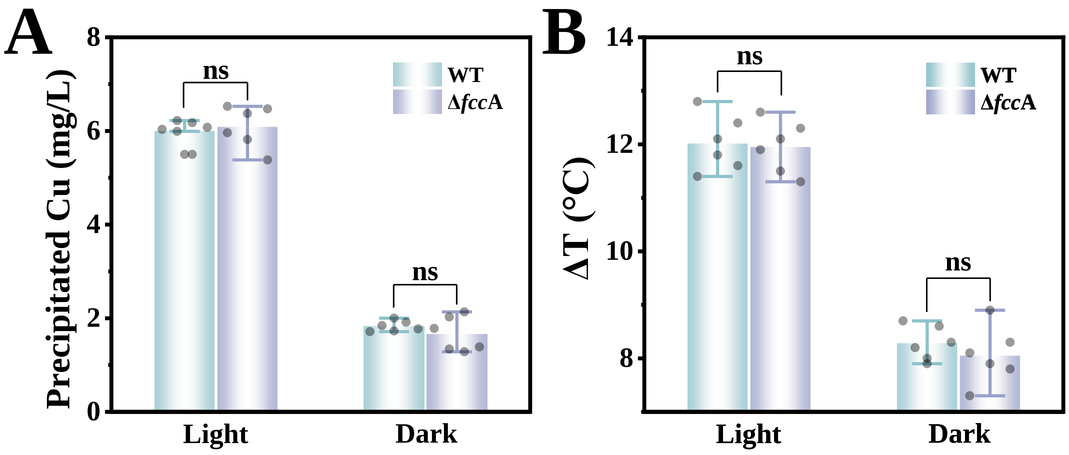

Supplementary Figure 7. The function of fumarate reductase FccA in $Cu_{2-x}Se$ NPs synthesis. (**A**) The precipitated copper concentration and (**B**) temperature rise (ΔT) of the lysed hybrids that were co-incubated with WT/Δ*fcc*A strains for 5 h with/without illumination. The above experiments were performed in the mineral salt medium with 20 mM sodium lactate. The data points represent six (n = 6) independent experiments for each experimental group and are displayed as mean ± standard deviation (SD). p values were determined by a one-way analysis of variance. “ns” indicates not significant ($p > 0.05$).

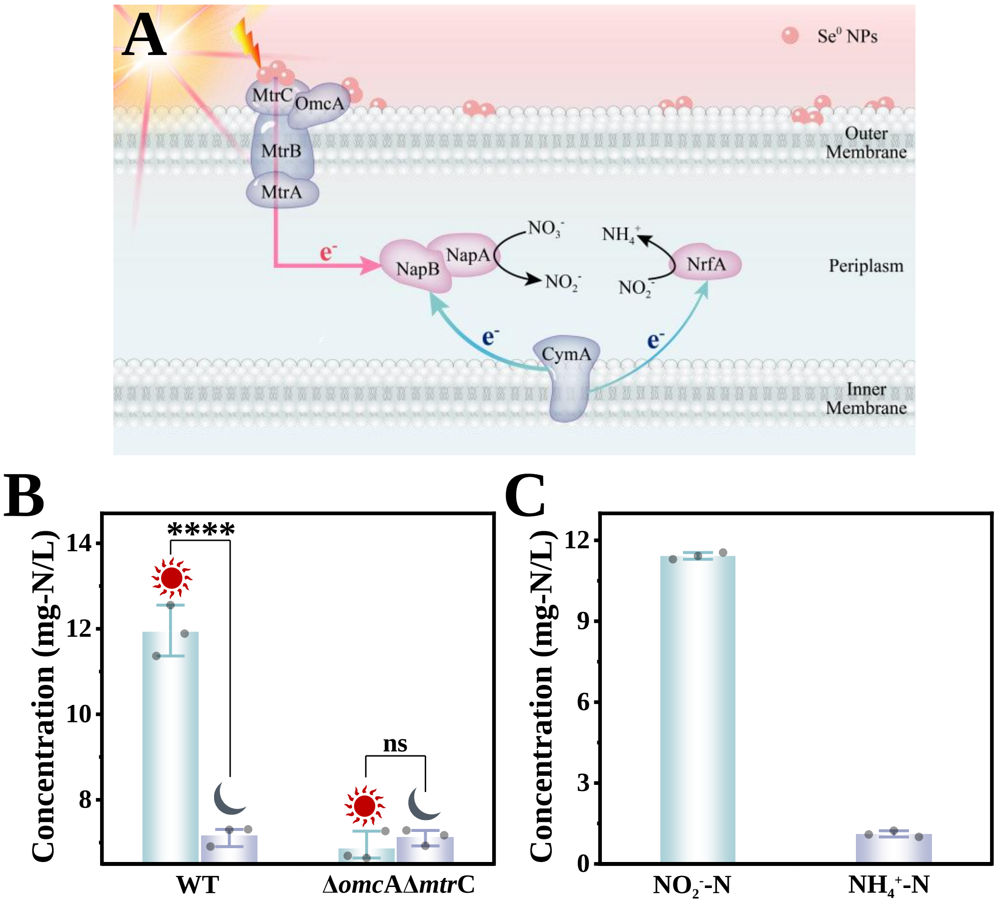

Supplementary Figure 8. Light-driven nitrate reduction by the *S. oneidensis*-Se^0 hybrid. (**A**) Proposed mechanism of the light-driven nitrate reduction by the *S. oneidensis*-Se^0 hybrid. (**B**) Concentrations of reduced nitrate (NO_3^--N) of the lysed hybrids that were co-incubated with WT/Δ*omc*AΔ*mtr*C and nitrate with/without illumination for 10 h. (**C**) Concentrations of produced nitrite (NO_2^--N) and ammonia (NH_4^+-N) of the lysed hybrids that were co-incubated with WT and nitrate with illumination for 10 h. The above experiments were performed in the mineral salt medium with 20 mM sodium acetate. The data points represented in (**B-C**) represent three (n = 3) independent experiments for each experimental group and are displayed as mean ± standard deviation (SD). p values of (**B**) were determined by a one-way analysis of variance. "****" represents $p < 0.0001$. "ns" indicates not significant ($p > 0.05$).

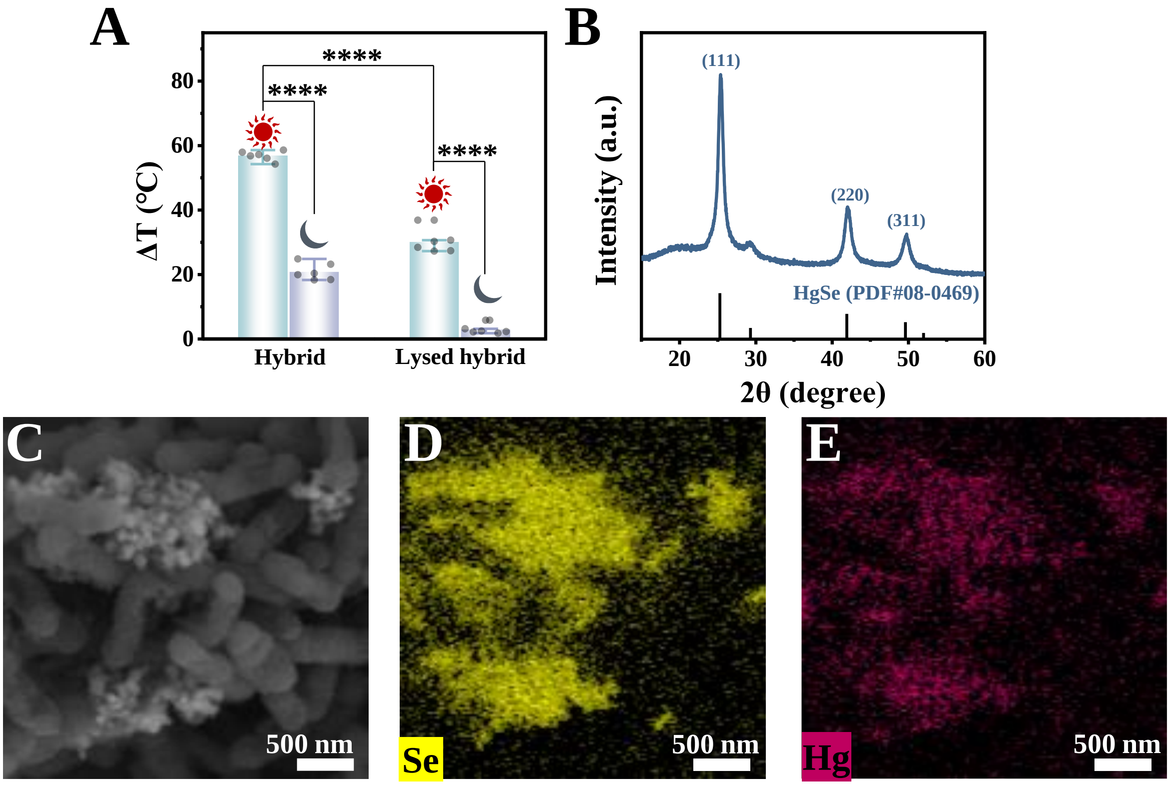

Supplementary Figure 9. Light-driven HgSe NPs assembly by the *S. oneidensis*-Se^0 hybrid and the characteristics of the biogenic HgSe NPs. (**A**) The precipitated mercury concentration by the *S. oneidensis*-Se^0 hybrid or cell-inactivated lysed hybrid with/without illumination. (**B**) XRD patterns of the formed *S. oneidensis*-HgSe NPs and standard HgSe (PDF#08-0469). (**C**) SEM image and the corresponding EDS mapping images of (**D**) Se and (**E**) Hg elements of the photosynthesized *S. oneidensis*-HgSe nanoparticles. The above experiments were performed in the mineral salt medium with 20 mM sodium lactate. The data points represented in (**A**) represent six (n = 6) independent experiments for each experimental group and are displayed as mean ± standard deviation (SD). p values of (**A**) were determined by a one-way analysis of variance. "****" represents p < 0.0001. Experiments of (**C**) were repeated three times with similar results.

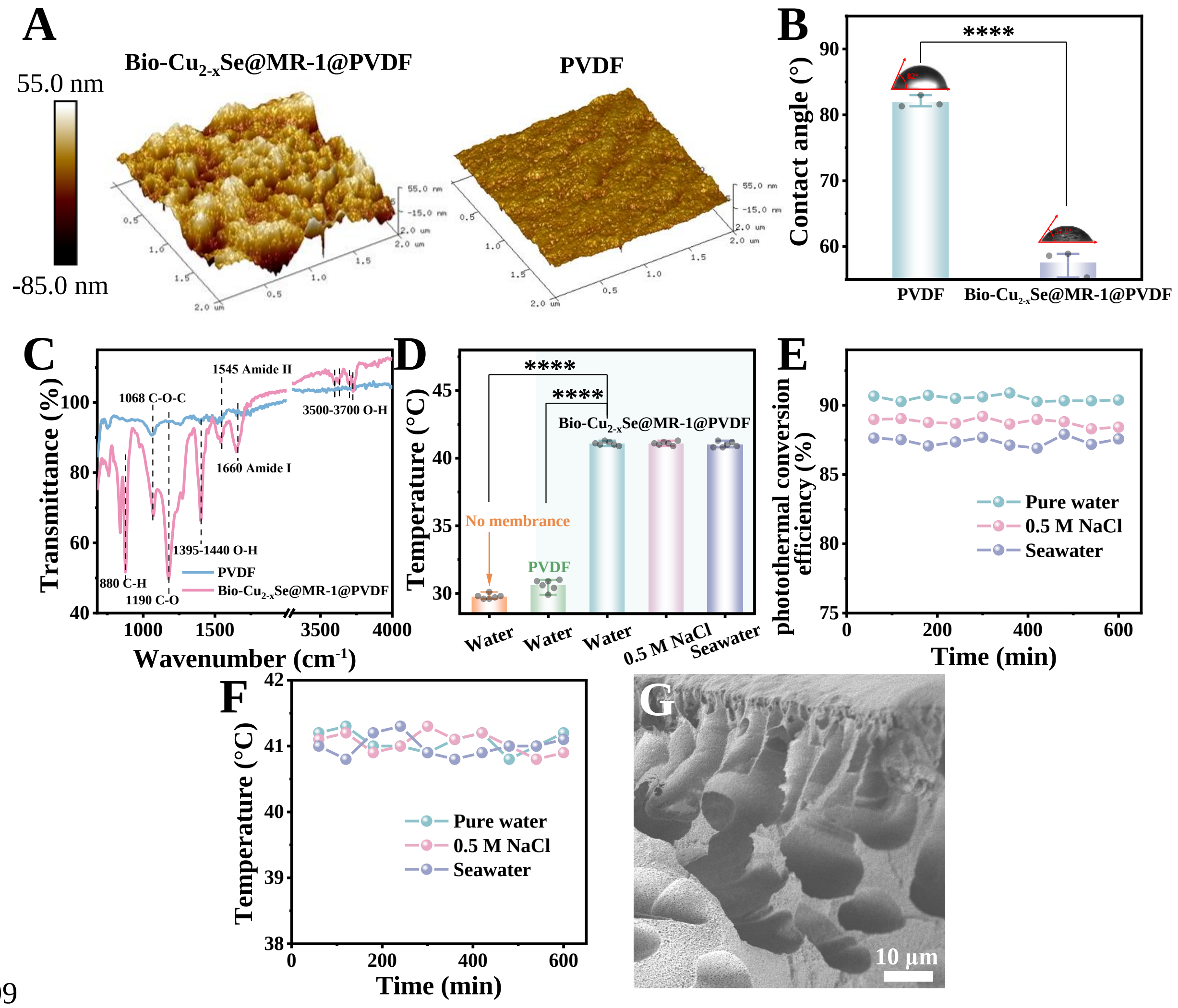

Supplementary Figure 10. Characteristics and corresponding solar vapor generation performance of the Bio-Cu_{2-x}Se@MR-1@PVDF and PVDF membranes. (**A**) 3D-AFM surface images of Bio-Cu_{2-x}Se@MR-1@PVDF and PVDF membranes. (**B**) Contact angle and (**C**) ATR-FTIR spectra of membranes. (**D**) Maximum temperature of surfaces for membranes under 1 sun irradiation after 1 h. (**E**) Photothermal conversion efficiency and (**F**) maximum temperature of surfaces for Bio-Cu_{2-x}Se@MR-1@PVDF membrane under 1 sun irradiation after 10 h of pure water, 0.5 M NaCl, and seawater. (**G**) SEM image of the cross-section of PVDF membrane. The data points represented in (**B**) represent three ($n = 3$) and in (**D**) represent six ($n = 6$) independent experiments for each experimental group and are displayed as mean ± standard deviation (SD). p values of (**B**) were determined by a independent samples t-test and (**D**) were determined by a one-way analysis of variance. "****" represents $p < 0.0001$. Experiments of (**G**) were repeated three times with similar results.

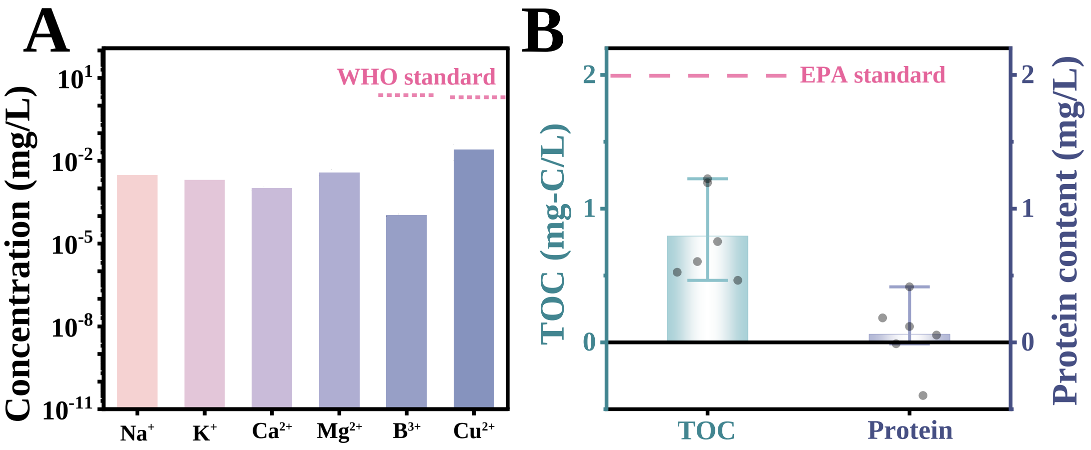

Supplementary Figure 11. The quality of distilled water produced by Bio-Cu_{2-x}Se@MR-1@PVDF membrane exposed to 1 sun for 10 h in seawater. (**A**) Concentrations of Na^{+}, K^{+}, Ca^{2+}, Mg^{2+}, B^{3+}, Cu^{2+} in distilled water. (**B**) TOC concentration and protein content in distilled water. The data points represented in (**B**) represent six (n = 6) independent experiments for each experimental group and are displayed as mean ± standard deviation (SD).

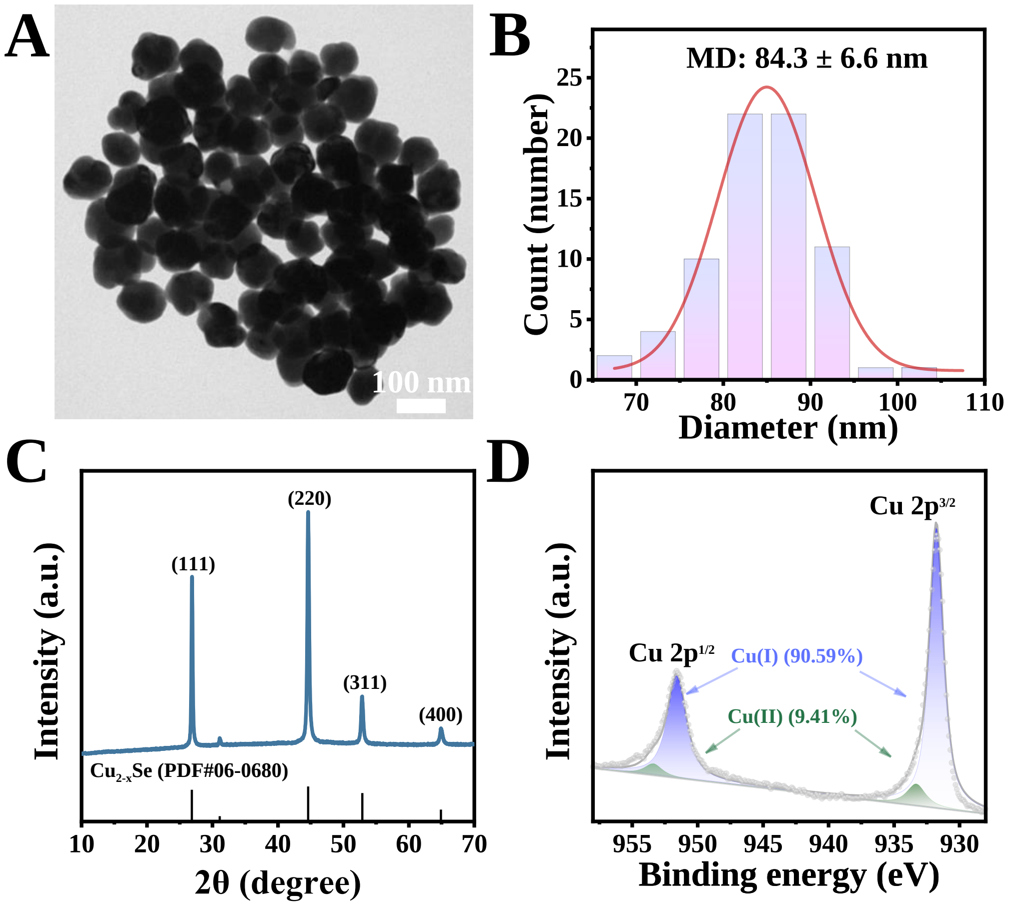

Supplementary Figure 12. Characteristics of the chemically synthesized $Cu_{2-x}Se$ nanoparticles (Chem-$Cu_{2-x}Se$ NPs). (**A**) TEM image of the Chem-$Cu_{2-x}Se$ NPs. (**B**) Average particle size of Chem-$Cu_{2-x}Se$ NPs. (**C**) XRD pattern of the Chem-$Cu_{2-x}Se$ NPs and standard $Cu_{2-x}Se$ (PDF#06-0680). (**D**) XPS spectrum with peak fitting for Cu 2*p*. Experiments of (**A**) were repeated three times with similar results.

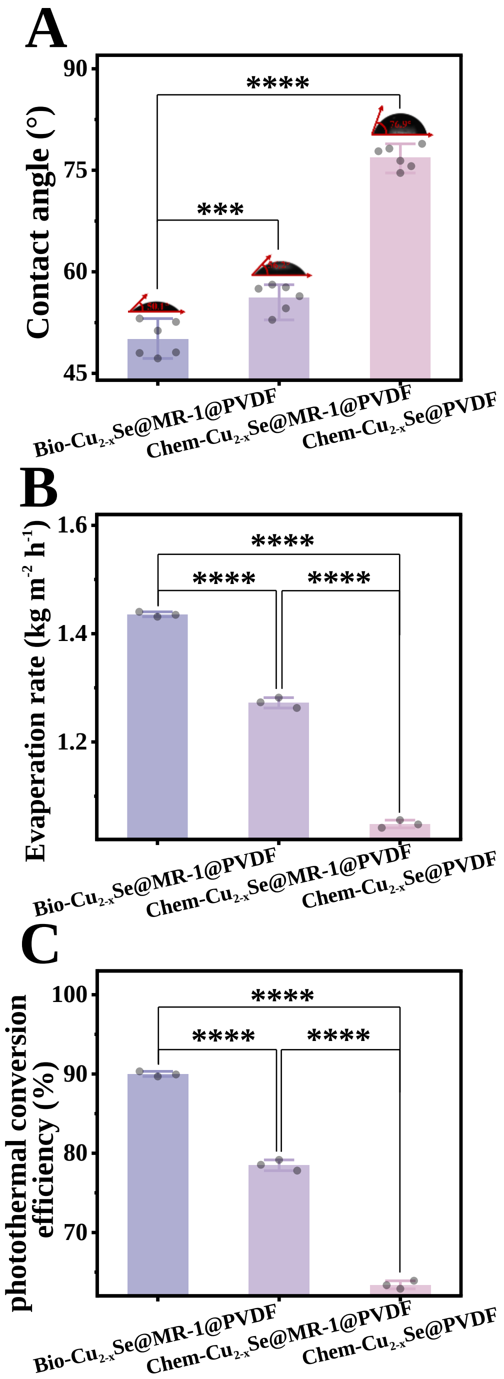

Supplementary Figure 13. Characteristics and corresponding solar vapor generation performance of the Bio-$Cu_{2-x}Se$@MR-1@PVDF, Chem-$Cu_{2-x}Se$@MR-1@PVDF and Chem-$Cu_{2-x}Se$@PVDF membranes. (**A**) Contact angle. (**B**) Evaporation rate under 1 sun irradiation. (**C**) Photothermal conversion efficiency under 1 sun irradiation. The data points represented in (**A**) represent six (n = 6) and in (**B-C**) represent three (n = 3) independent experiments for each experimental group and are displayed as mean ± standard deviation (SD). p values were determined by a one-way analysis of variance. "****" represents $p < 0.0001$. "***" represents $p < 0.001$.

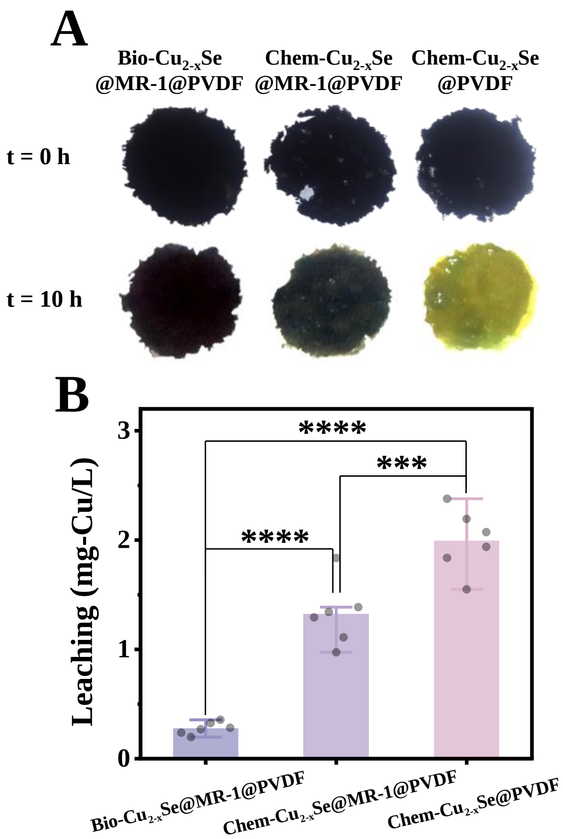

Supplementary Figure 14. Stability of the Bio-Cu_{2-x}Se@MR-1@PVDF, Chem-Cu_{2-x}Se@MR-1@PVDF and Chem-Cu_{2-x}Se@PVDF membranes exposed to 1 sun. (**A**) Optical pictures of different membranes before/after 10 h photothermal distillation experiments. (**B**) The Cu leaching concentration in the original water body of different membranes exposed to 1 sun for 10 h of pure water. The data points represented in (**B**) represent six (n = 6) independent experiments for each experimental group and are displayed as mean ± standard deviation (SD). p values were determined by a one-way analysis of variance. "****" represents $p < 0.0001$. "***" represents $p < 0.001$.

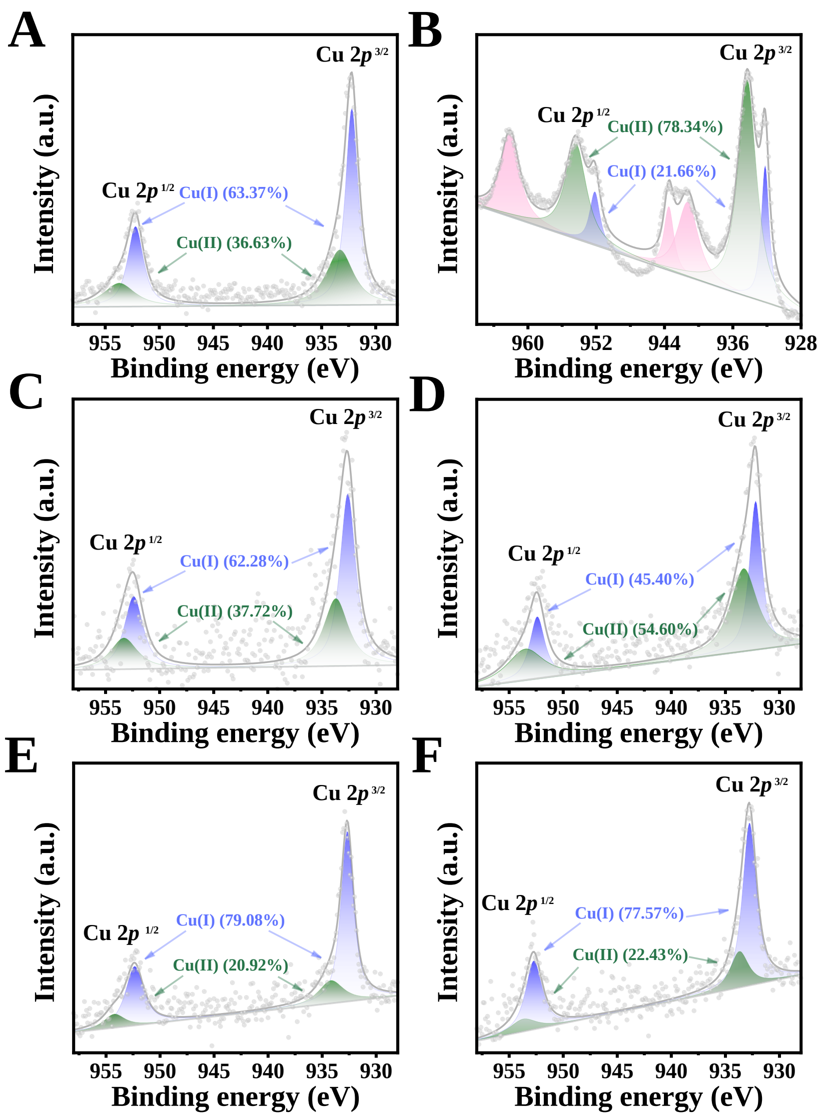

Supplementary Figure 15. XPS results of different membranes before (A, C and E) / after (B, D and F) the photothermal distillation experiments exposed to 1 sun for 10 h. XPS spectra with peak fitting of Cu 2*p* for (**A, B**) Chem-$Cu_{2-x}Se$@PVDF, (**C**, **D**) Chem-$Cu_{2-x}Se$@MR-1@PVDF and (**E**, **F**) Bio-$Cu_{2-x}Se$@MR-1@PVDF membranes.

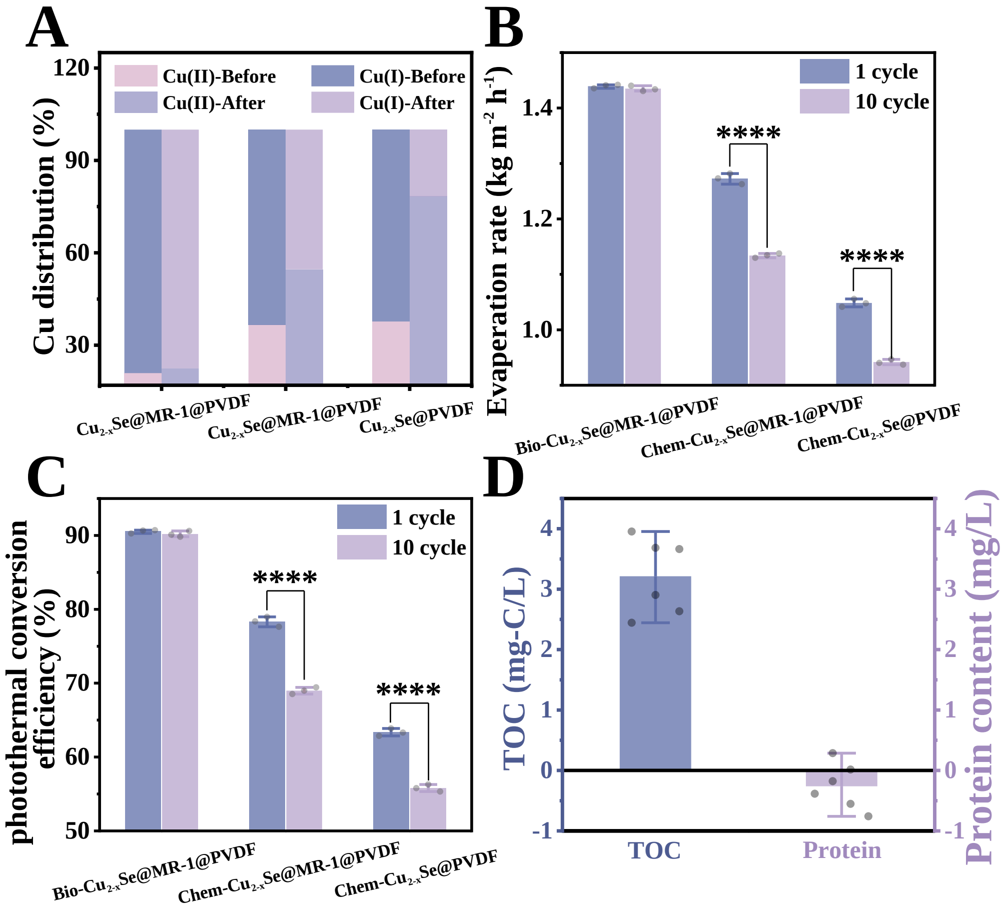

Supplementary Figure 16. Characterization of the stability of different membranes. (**A**) The proportion of Cu(II) or Cu(I) in the membranes before/after exposing to 1 sun irradiation for 10 h. (**B**) The evaporation rate of membranes before/after exposing to 1 sun irradiation for 10 h. (**C**) The photothermal conversion efficiency of membranes before/after exposing to 1 sun irradiation for 10 h. (**D**) TOC concentration and protein content in the original water body of Bio-$Cu_{2-x}Se$@MR-1@PVDF membrane exposed to 1 sun for 10 h. The data points represented in (**A-B**) represent three (n = 3) and in (**C**) represent six (n = 6) independent experiments for each experimental group and are displayed as mean ± standard deviation (SD). p values of (**B-C**) were determined by a one-way analysis of variance. "****" represents $p < 0.0001$.

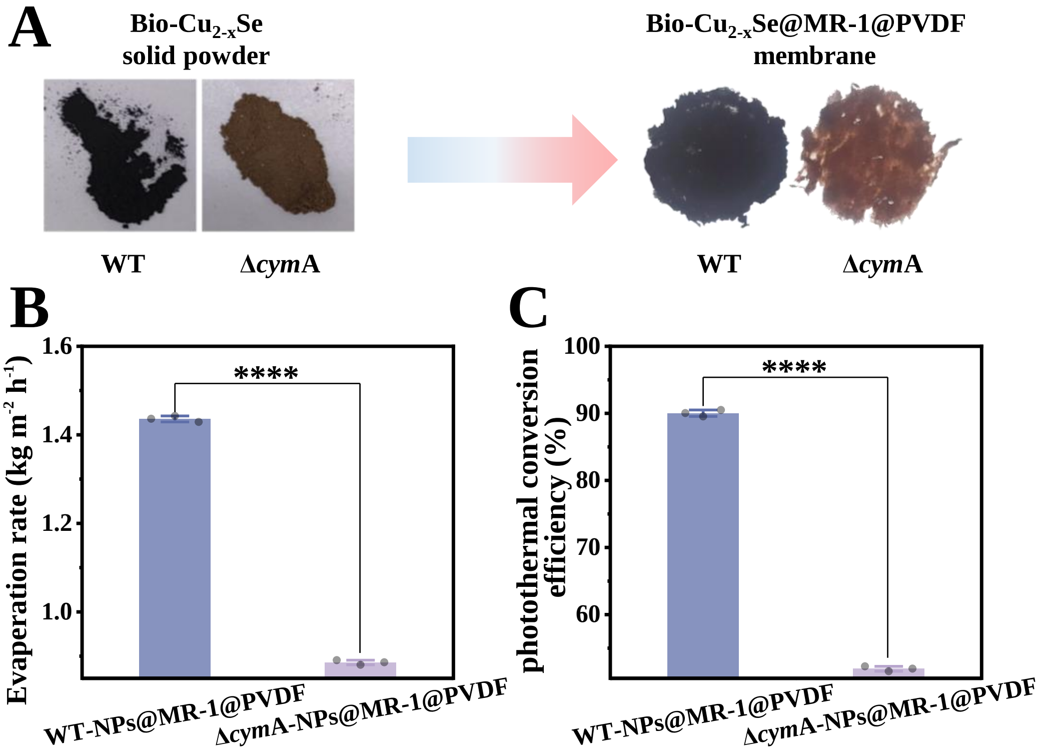

Supplementary Figure 17. Solar vapor generation performance of Bio-Cu_{2-x}Se@MR-1@PVDF membrane (using WT as bio-nano-factory) and Δ*cym*A-NPs@MR-1@PVDF (using Δ*cym*A as bio-nano-factory). (**A**) Schematic diagram of the Bio-Cu_{2-x}Se of solid powder and corresponding Bio-Cu_{2-x}Se@MR-1@PVDF membrane. (**B**) Evaporation rate of membranes under 1 sun irradiation. (**C**) Photothermal conversion efficiency of membranes under 1 sun irradiation. The synthetic precursors of biogenic Cu_{2-x}Se were all lysed hybrid-Se^0 NPs. The data points represented in (**B-C**) represent three (n = 3) independent experiments for each experimental group and are displayed as mean ± standard deviation (SD). p values were determined by an independent samples t-test. "****" represents $p < 0.0001$.

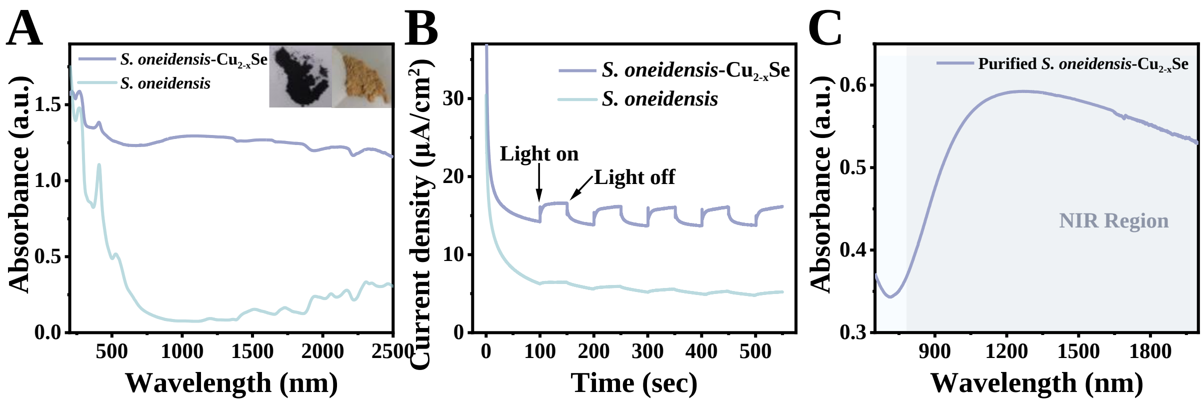

Supplementary Figure 18. Properties of the biogenic $Cu_{2-x}Se$ NPs. (**A**) UV-vis DRS spectra of the *S. oneidensis*-$Cu_{2-x}Se$ and *S. oneidensis* control and the corresponding optical pictures (left inset: *S. oneidensis*-$Cu_{2-x}Se$. right inset: *S. oneidensis*). (**B**) *I-t* curves of the *S. oneidensis*-$Cu_{2-x}Se$ and *S. oneidensis* control with a light on/off cycle (50/50 s). (**C**) UV-vis-NIR absorption spectrum of purified $Cu_{2-x}Se$ NPs (in CCl_4).

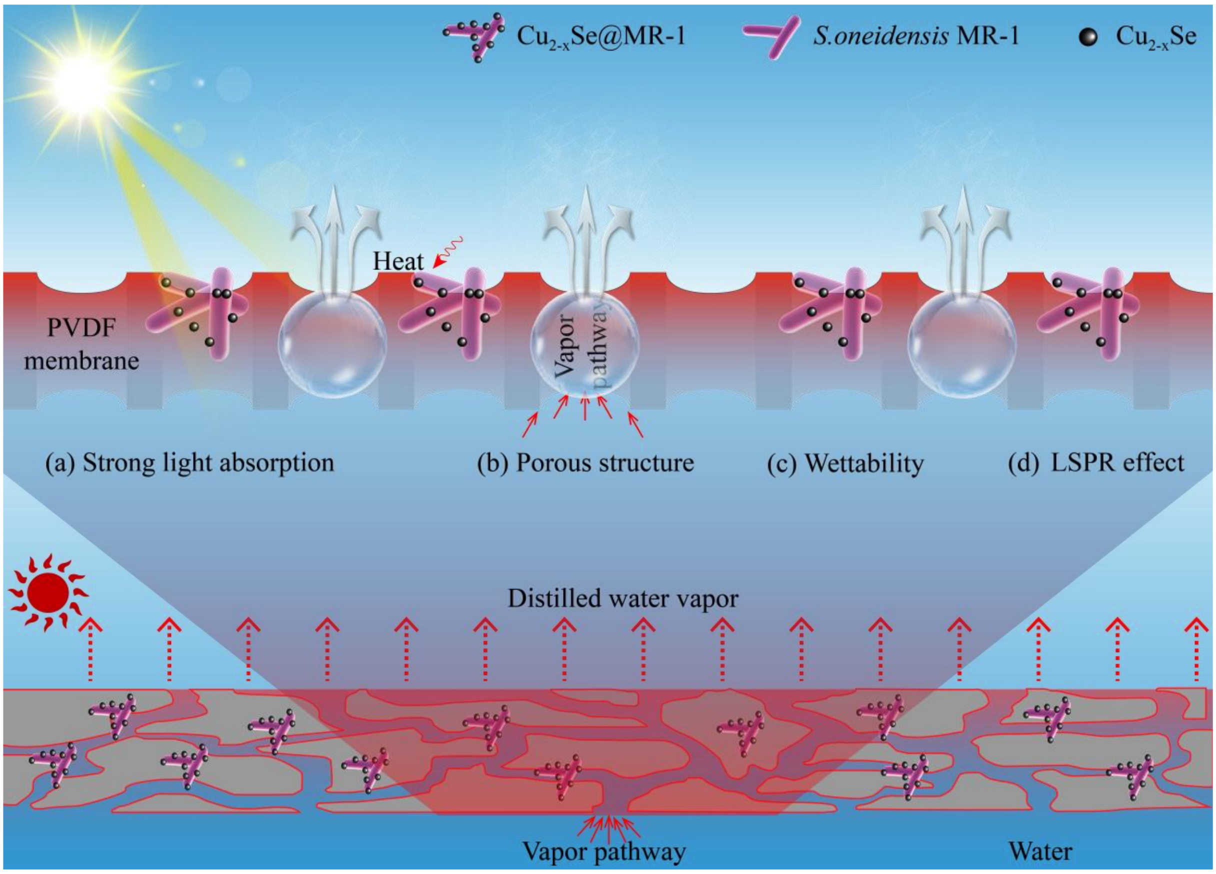

Supplementary Figure 19. Schematic illustration of the working mechanism of Bio-$Cu_{2-x}Se$@MR-1@PVDF membrane in the water desalination process.

Supplementary references

1. Han, H.X. et al. Reversing electron transfer chain for light-driven hydrogen production in botic-abiotic hybrid systems. *J. Am. Chem. Soc.* **144**, 6434-6441 (2022).
2. Zhang Y., W.P., Li H., Luo H. J., Luo W. H., Lin Z. X. Determination of cuprous in biological samples by liquid-liquid extraction-GFAAS. *Spectrosc. Spect. Anal.* **40**, 632-636 (2020).
3. Chen, D. et al. An improved bathocuproine assay for accurate valence identification and quantification of copper bound by biomolecules. *Anal. Biochem.* **497**, 27-35 (2016).
4. E.W. Rice, R.B.B., A.D. Eaton Standard methods for the examination of water and wastewater. *Am. Phys. Educ. Rev.* **24**, (9) (1995).
5. Zhang, S. et al. Vacancy engineering of $Cu_{2-x}Se$ nanoparticles with tunable LSPR and magnetism for dual-modal imaging guided photothermal therapy of cancer. *Nanoscale* **10**, 3130-3143 (2018).
6. Chen, M. et al. Plasmonic nanoparticle-embedded poly(p-phenylene benzobisoxazole) nanofibrous composite films for solar steam generation. *Nanoscale* **10**, 6186-6193 (2018).
7. Liu, Y. et al. A bioinspired, reusable, paper-based system for high-performance large-scale evaporation. *Adv. Mater.* **27**, 2768-2774 (2015).
8. Zhou, X., Zhao, F., Guo, Y., Zhang, Y. & Yu, G. A hydrogel-based antifouling solar evaporator for highly efficient water desalination. *Energy Environ. Sci.* **11**, 1985-1992 (2018).
9. Li, Y. et al. 3D-printed, all-in-one evaporator for high-efficiency solar steam generation under 1 sun illumination. *Adv. Mater.* **29** (2017).
10. Wang, G. et al. Reusable reduced graphene oxide based double-layer system modified by polyethylenimine for solar steam generation. *Carbon* **114**, 117-124 (2017).
11. Fu, Y. et al. Oxygen plasma treated graphene aerogel as a solar absorber for rapid and

efficient solar steam generation. *Carbon* **130**, 250-256 (2018).

12. Shi, L., Wang, Y., Zhang, L. & Wang, P. Rational design of a bi-layered reduced graphene oxide film on polystyrene foam for solar-driven interfacial water evaporation. *J. Mater. Chem. A* **5**, 16212-16219 (2017).
13. Liang, H. et al. Thermal efficiency of solar steam generation approaching 100 % through capillary water transport. *Angew Chem. Int. Ed. Engl.* **58**, 19041-19046 (2019).
14. Yu, Z., Cheng, S., Li, C., Li, L. & Yang, J. Highly efficient solar vapor generator enabled by a 3D hierarchical structure constructed with hydrophilic carbon felt for desalination and wastewater treatment. *ACS Appl. Mater. Interfaces* **11**, 32038-32045 (2019).
15. Zhao, F. et al. Highly efficient solar vapour generation via hierarchically nanostructured gels. *Nat. Nanotechnol.* **13**, 489-495 (2018).
16. Kaur, M., Ishii, S., Shinde, S.L. & Nagao, T. All-ceramic microfibrous solar steam generator: TiN plasmonic nanoparticle-loaded transparent microfibers. *ACS Sustainable Chem. Eng.* **5**, 8523-8528 (2017).
17. Cheng, H. et al. Tailoring core@shell structure of $Cu_{2-x}Se$@PDAs for synergistic solar-driven water evaporation. *J. Mater. Sci.* **57**, 11725-11734 (2022).
18. Xia, W., Cheng, H., Zhou, S., Yu, N. & Hu, H. Synergy of copper selenide/MXenes composite with enhanced solar-driven water evaporation and seawater desalination. *J. Colloid. Interface Sci.* **625**, 289-296 (2022).
19. Tao, F. et al. A plasmonic interfacial evaporator for high-efficiency solar vapor generation. *Sustain. Energy Fuels* **2**, 2762-2769 (2018).
20. Tao, F. et al. Copper sulfide-based plasmonic photothermal membrane for high-efficiency solar vapor generation. *ACS Appl. Mater. Interfaces* **10**, 35154-35163 (2018).
21. Guo, Z. et al. Super-hydrophilic copper sulfide films as light absorbers for efficient solar steam generation under one sun illumination. *Semicond. Sci. Technol.* **33**, 025008 (2018).
22. Qin, Y. et al. Dyeable PAN/CuS nanofiber membranes with excellent mechanical and

photothermal conversion properties via electrospinning. *ACS Appl. Polym. Mater.* **4**, 9144-9150 (2022).

23. Shang, M. et al. Full-spectrum solar-to-heat conversion membrane with interfacial plasmonic heating ability for high-efficiency desalination of seawater. *ACS Appl. Energy Mater.* **1**, 56-61 (2017).
24. Wang, Z., Zhang, X.F., Shu, L. & Yao, J. Copper sulfide integrated functional cellulose hydrogel for efficient solar water purification. *Carbohydr. Polym.* **319**, 121161 (2023).
25. Chen, J. et al. Photothermal membrane of CuS/polyacrylamide-carboxymethyl cellulose for solar evaporation. *ACS Appl. Polym. Mater.* **3**, 2402-2410 (2021).
26. Lampis, S. et al. Selenite biotransformation and detoxification by *Stenotrophomonas maltophilia* SeITE02: Novel clues on the route to bacterial biogenesis of selenium nanoparticles. *J. Hazard. Mater.* **324**, 3-14 (2017).
27. Tugarova, A.V., Mamchenkova, P.V., Dyatlova, Y.A. & Kamnev, A.A. FTIR and Raman spectroscopic studies of selenium nanoparticles synthesised by the bacterium *Azospirillum thiophilum*. *Spectrochim Acta A: Mol. Biomol. Spectrosc.* **192**, 458-463 (2018).
28. Tam, K. et al. Growth mechanism of amorphous selenium nanoparticles synthesized by *Shewanella* sp. HN-41. *Biosci. Biotechnol. Biochem.* **74**, 696-700 (2010).
29. Ho, C.T. et al. Biogenic synthesis of selenium nanoparticles by *Shewanella* sp. HN-41 using a modified bioelectrochemical system. *Electronic J. Biotechnol.* **54**, 1-7 (2021).
30. Beleneva, I.A. et al. Biogenic synthesis of selenium and tellurium nanoparticles by marine bacteria and their biological activity. *World J. Microbiol. Biotechnol.* **38**, 188 (2022).
31. Zhang, X., Zhong, M., Zhou, R., Qin, W. & Si, Y. Se(IV) reduction and extracellular biosynthesis of Nano-Se(0) by *Shewanella oneidensis* MR-1 and *Shewanella putrefaciens*. *Process Biochem.* **130**, 481-491 (2023).
32. Tian, L.J. et al. Directed biofabrication of nanoparticles through regulating extracellular electron transfer. *J. Am. Chem. Soc.* **139**, 12149-12152 (2017).

33. Li, D.B. et al. Selenite reduction by *Shewanella oneidensis* MR-1 is mediated by fumarate reductase in periplasm. *Sci. Rep.* **4**, 3735 (2014).

34. Qi, S. et al. Extracellular biosynthesis of $Cu_{2-x}Se$ nanocrystallites with photocatalytic activity. *Materi. Res. Bull.* **111**, 126-132 (2019).

35. Wang, X.-M. et al. Highly efficient near-infrared photothermal antibacterial membrane with incorporated biogenic CuSe nanoparticles. *Chem. Eng. J.* **405** (2021).

36. Wang, X.M. et al. AQDS activates extracellular synergistic biodetoxification of copper and selenite via altering the coordination environment of outer-membrane proteins. *Environ. Sci. Technol.* **56**, 13786-13797 (2022).

37. Wang, X.M. et al. Biogenic copper selenide nanoparticles for near-infrared photothermal therapy application. *ACS Appl. Mater. Interfaces* **15**, 27638-27646 (2023).

38. Ross, D.E., Flynn, J.M., Baron, D.B., Gralnick, J.A. & Bond, D.R. Towards electrosynthesis in *Shewanella*: energetics of reversing the mtr pathway for reductive metabolism. *PLoS One* **6**, e16649 (2011).

39. Rowe, A.R. et al. Tracking electron uptake from a cathode into *Shewanella* cells: implications for energy acquisition from solid-substrate electron donors. *mBio* **9**, e02203-02217 (2018).

40. Tefft, N.M. & TerAvest, M.A. Reversing an extracellular electron transfer pathway for electrode-driven acetoin reduction. *ACS Synth. Biol.* **8**, 1590-1600 (2019).

41. Li, Y. et al. Microbial electrosynthetic nitrate reduction to ammonia by reversing the typical electron transfer pathway in *Shewanella oneidensis*. *Cell Reports Physical Science* **4**, 101433 (2023).

42. Shen, H. et al. A whole-cell inorganic-biohybrid system integrated by reduced graphene oxide for boosting solar hydrogen production. *ACS Catal.* **10**, 13290-13295 (2020).

43. Xiao, X. et al. Anaerobically photoreductive degradation by CdS nanocrystal: Biofabrication process and bioelectron-driven reaction coupled with *Shewanella*

oneidensis MR-1. *Biochem. Eng. J.* **154**, 107466 (2020).

44. Zhou, E. et al. Direct microbial electron uptake as a mechanism for stainless steel corrosion in aerobic environments. *Water Res.* **219**, 118553 (2022).
45. Huang, B.C., Yi, Y.C., Chang, J.S. & Ng, I.S. Mechanism study of photo-induced gold nanoparticles formation by *Shewanella oneidensis* MR-1. *Sci. Rep.* **9**, 7589 (2019).
46. Lintern, M., Anand, R., Ryan, C. & Paterson, D. Natural gold particles in Eucalyptus leaves and their relevance to exploration for buried gold deposits. *Nat. Commun.* **4**, 2614 (2013).
47. Tian, L.-J. et al. Bio-assembly of CdS_xSe_{1-x} quantum dots in ryegrass. *Green Chem.* **21**, 6727-6730 (2019).
48. Zhu, X. et al. Photosynthesis-mediated intracellular biomineralization of gold nanoparticles inside Chlorella cells towards hydrogen boosting under green light. *Angew Chem. Int. Ed.*, e202308437 (2023).
49. Hanna, A.L. et al. Biosynthesis and characterization of silver nanoparticles produced by phormidium ambiguum and desertifilum tharense cyanobacteria. *Bioinorg. Chem. Appl.* **2022**, 9072508 (2022).
50. Guo, J. et al. Light-driven fine chemical production in yeast biohybrids. *Science* **362**, 813-816 (2018).
51. Zhang, H. et al. Bacteria photosensitized by intracellular gold nanoclusters for solar fuel production. *Nat. Nanotechnol.* **13**, 900-905 (2018).
52. Sakimoto, K.K., Wong, A.B. & Yang, P. Self-photosensitization of nonphotosynthetic bacteria for solar-to-chemical production. *Science* **351**, 74-77 (2016).
53. Lin, Y. et al. Periplasmic biomineralization for semi-artificial photosynthesis. *Sci. Adv.* **9**, eadg5858 (2023).
54. Ye, J. et al. Solar-driven methanogenesis with ultrahigh selectivity by turning down H_2 production at biotic-abiotic interface. *Nat. Commun.* **13**, 6612 (2022).
55. Wei, W. et al. A surface-display biohybrid approach to light-driven hydrogen production in

air. *Sci. Adv.* **4**, eaap9253 (2018).

56. Martins, M., Toste, C. & Pereira, I.A.C. Enhanced light-driven hydrogen production by self-photosensitized biohybrid systems. *Angew Chem. Int. Ed.* **60**, 9055-9062 (2021).
57. Luo, B.F. et al. A periplasmic photosensitized biohybrid system for solar hydrogen production. *Adv. Energy Mater.* **11**, 2100256 (2021).
